# Emotional Evaluations from Partners and Opponents Differentially Influence the Perception of Ambiguous Faces

**DOI:** 10.3390/bs14121168

**Published:** 2024-12-05

**Authors:** Danyang Ran, Yihan Zhang, Bin Hao, Shuaixia Li

**Affiliations:** 1 Research Center of Brain and Cognitive Neuroscience, Liaoning Normal University, Dalian 116029, China; rrddyy1234567@163.com (D.R.); zyhnk2009@sina.com (Y.Z.); hh943902190@163.com (B.H.); 2 Key Laboratory of Brain and Cognitive Neuroscience, Liaoning Province, Dalian 116029, China

**Keywords:** contextual valence, interpersonal distance, surprised face, mass univariate statistics, ERPs

## Abstract

The influence of contextual valence and interpersonal distance on facial expression perception remains unclear despite their significant role in shaping social perceptions. In this event-related potential (ERP) study, we investigated the temporal dynamics underlying the processing of surprised faces across different interpersonal distances (partner, opponent, or stranger) and contextual valence (positive, neutral, or negative) contexts. Thirty-five participants rated the valence of surprised faces. An advanced mass univariate statistical approach was utilized to analyze the ERP data. Behaviorally, surprised faces in partner-related negative contexts were rated more negatively than those in opponent- and stranger-related contexts. The ERP results revealed an increased P1 amplitude for surprised faces in negative relative to neutral contexts. Both the early posterior negativity (EPN) and late positive potentials (LPP) were also modulated by contextual valence, with larger amplitudes for faces in positive relative to neutral and negative contexts. Additionally, when compared to stranger-related contexts, faces in partner-related contexts exhibited enhanced P1 and EPN responses, while those in opponent-related contexts showed amplified LPP responses. Taken together, these findings elucidate the modulation of intricate social contexts on the perception and interpretation of ambiguous facial expressions, thereby enhancing our understanding of nonverbal communication and emotional cognition.

## 1. Introduction

Facial expressions serve as essential cues for deciphering the emotions and intentions of others and thus play a pivotal role in interpersonal communication [1]. However, the perception of facial expressions in everyday life is heavily affected by both emotional and non-emotional contextual information. [2,3,4]. The available evidence converges to support the notion that various emotional contexts, including emotional facial expressions [5,6], verbal sentences [7,8], scene pictures [1,9,10,11], body expressions [12,13], and other types of stimuli [14,15], exert a powerful impact on facial expression perception. Interestingly, individuals could precisely infer the affective state of a target person even in the absence of visible facial expressions. This was achieved solely by relying on contextual information [16,17]. Regarding the facial expressions conveying clear valence, a common finding is that contextual cues can either facilitate or inhibit the recognition of these expressions. This effect depends on the emotional congruence between the face and context [12,18,19].

In fact, individuals frequently encounter facial expressions with ambiguous valence in interpersonal communication [20]. The findings of recent studies indicate that neutral and surprised faces are perceived as more pleasant in positive contexts and more unpleasant in negative ones [8,21,22,23], highlighting their context-dependent nature [24]. Moreover, Baum and Abdel Rahman [25] proposed that the emotional context effect of these faces is immune to the contextual credibility. Furthermore, factors such as social relevance [26,27,28], feedback source [29,30], and self-reference [23,31,32,33] also impact ambiguous face processing. For instance, Wieser et al. [8] investigated the perception of neutral faces in self-referential postive/negative contexts and found that neutral faces in a self-related context were perceived as more pleasant/unpleasant as compared to other-related context. Similar findings were observed for surprised faces [23]. These observations suggest that complex social contexts synergistically shape the perception of ambiguous faces.

The electroencephalogram (EEG), the electrical signal recorded from the scalp that reflects the activity of populations of brain cells as they fire, is one of the most versatile and informative signals used in the field of neuroscience. Once EEG signals are obtained, event-related potentials (ERPs) are extracted, and various analytical methods are employed to explore the information they contain [34]. Electrophysiological evidence has revealed that several ERP components indexing distinct cognitive processes, such as the P1, N170, early posterior negativity (EPN), and late positive potentials (LPP) components, are modulated by both faces and contextual information. Specifically, P1, a component that peaks at approximately 100–130 ms over the temporo-occipital sites after stimulus onset, is believed to possess sensitivity towards low-level physical characteristics of visual stimuli [35]. Some studies have reported increased P1 amplitudes in response to fearful faces, reflecting an early automatic negativity bias [36,37,38]. Other research suggests that contextual information modulates the P1 response to target faces [10,15,39]. For example, Li et al. [40] demonstrated that surprised faces in fearful contexts elicited a larger P1 amplitude than those in happy contexts, suggesting an early attentional bias towards threatening face-context information. However, other studies failed to show a significant P1 modulation by emotional faces [41,42] or face-related contexts [22,23].

The N170 component is believed to reflect structural face encoding [43,44]. There is ample evidence that faces expressing anger and fear produce larger N170 amplitudes than neutral and happy faces across various manipulations of images [41,45,46], task demands [47,48,49,50], and attentional resources [51,52], reflecting the automatic processing of threats. Furthermore, Righart and colleagues found that fearful faces presented in fearful contexts elicited larger N170 amplitudes compared to those in happy and neutral contexts [10,11]. Similarly, some previous studies showed a significantly enhanced N170 response to surprised faces in negative relative to neutral contexts [23,53]. These findings suggest that the N170 component is crucial in integrating face-context information and is especially susceptible to threatening information.

The EPN is typically associated with early selective attention and strategic encoding of emotional stimuli [29,54,55,56]. Numerous studies have revealed that threatening [47,50,55] and happy faces [57,58] can amplify the EPN responses compared to neutral faces, reflecting the sensitivity to emotional arousal. Additionally, the EPN elicited by neutral faces is enhanced when presented in a negative relative to neutral context [8,22]. Similarly, the self-referential context can also modulate the EPN, with faces presented in self-related contexts eliciting larger amplitudes compared to those in other-related contexts [8,33]. More importantly, McCrackin and Itier [32] proposed that contextual valence and self-reference can modulate the EPN interactively. This is supported by the observation of enlarged amplitudes for neutral faces in self-related positive contexts relative to other-related positive contexts, indicating the involvement of self-positivity bias.

The LPP is functionally linked to the elaborated processing of and sustained attention to emotional stimuli [55,59,60]. In comparison to neutral faces, fearful and happy faces are consistently found to increase LPP amplitudes [38,52]. Apart from the emotional relevance of a face per se, this component is strongly modulated by contextual information in a top-down manner [21,54,61]. More relevant to the present study, surprised or neutral faces have been found to increase the LPP amplitudes when presented in positive and negative contexts [53,62]. Furthermore, it could vary as a function of contextual self-relevance, with increased responses to self-related faces relative to other-related faces [23,54].

Current research on the self-referential effect in facial expression perception has predominantly focused on discerning the distinctions between conditions related to oneself and those pertaining to others. However, as self-reference spans a dynamic continuum from weak to strong, the effect of its degree on the processing of facial expressions is still unclear. Furthermore, face perception is typically multidimensional, and there is an ongoing debate on whether contextual valence and self-reference can interactively shape face perception. Previously, we found that contextual valence and self-reference impacted the EPN interactively [23], but Wieser et al. [8] did not oberserve such an interaction in the processing of neutral faces. It is worth noting that the emotional sentences related to others in our study specifically described the target faces, whereas those in Wieser’s study pertained to irrelevant strangers. This inconsistency may stem from differences in the level of self-relevance. The perception of interpersonal distance often plays a significant role in shaping our behavioral responses during social interactions. Interpersonal distance refers to how closely individuals perceive others (e.g., partners, opponents, and strangers) in relation to themselves, encompassing thoughts, emotions, and behaviors [63,64], which involves varying degrees of self-relevance and can help elucidate the intricate contextual effects on face perception. Individuals experience a diverse range of competitive or cooperative occasions throughout their lives, but very few researchers have investigated the face perception in these contexts [27,28,65]. Balas and Thomas [28] stated that individuals perceive neutral faces as more aggressive in competitive contexts. Bublatzky et al. [54] also found enhanced LPP activity in response to happy, rather than angry or neutral, faces of the partners with whom participants will interact in the future. Although these findings indicate that competitive and cooperative contexts may shape face processing, the neural correlates underlying such effects remain poorly understood.

The objective of the present study was to explore the temporal dynamics of how emotional judgements in cooperative and competitive settings influence the perception of surprised faces. According to previous studies [8,23,33], we utilized verbal sentences to depict contextual valence and interpersonal distance as contexts. Participants were instructed to assess the affective valence of surprised faces. We predicted that surprised faces within partner-related negative contexts would be perceived more negatively, whereas those in positive contexts would be perceived as more positive [66]. At the electrophysiological level, we expected significant modulations in the P1, N170, EPN, and LPP components. Considering the P1’s sensitivity to threatening contexts [40], we hypothesized that surprised faces in negative contexts would elicit enhanced P1 amplitudes compared to positive and neutral ones. Building upon prior findings that showed larger N170 amplitudes for surprised faces following negative contexts, we anticipated a replicated pattern in this study [23]. Based on the self-positivity bias [23,32], we predicted larger EPN amplitudes for surprised faces in positive contexts than in neutral and negative contexts. As prior research showed remarkable sensitivity of the LPP to emotional and self-related contexts [23,53,54,62], we hypothesized that surprised faces in emotional or partner-related contexts would elicit larger LPP responses. Lastly, the interaction between interpersonal distance and contextual valence was approached from an exploratory perspective.

## 2. Materials and Methods

### 2.1. Participants

Forty healthy students from Liaoning Normal University participated in the experiment as paid volunteers and provided written informed consent. Due to substandard participant behavior and poor EEG data quality, five participants were discarded from the analysis, leaving a final sample of 35 participants (16 females; aged 18–27 years, mean age = 22.11 years, SD = 3.29). The sample size was determined to be sufficient for detecting a medium effect size (Cohen *f* = 0.25, power = 95%, α error = 0.05), based on a pre-study power analysis conducted using G*Power 3.1 software [67]. All participants were right-handed, with normal or corrected-to-normal vision, and reported no history of neurological or psychiatric disorders. The research was approved by the Ethics Committee of Liaoning Normal University (LL2024126).

### 2.2. Stimuli

A collection of 36 Chinese faces (half female) displaying surprised expressions was chosen from the Chinese Facial Affective Picture System (CFAPS) [68]. The average emotional intensity of these faces was 5.73 ± 0.55 (M ± SD) [68], with a mean identification rate of 84.35%. Using Adobe Photoshop CS6, each picture was resized to 480 pixels in length and 371 pixels in width, resulting in a visual angle of 8.0° × 10.3° when viewed at a distance of 70 cm from the computer screen. All the pictures were consistent in low-level physical features, such as background, brightness, and contrast level.

For context stimuli, based upon previous research [23], 144 descriptive sentences were generated, each incorporating contextual valence (positive, neutral, negative) and interpersonal distance (partner, opponent, and stranger) conditions, with 24 sentences in each condition. All sentences were formatted using the same font (KaiTi, Regular) and size (30 pixels) and were meticulously matched for word length and grammatical structure. In a pilot study, a separate sample of 20 participants (15 females; aged 21–30 years; mean age = 23.3 years) were asked to assess the valence and arousal of these sentences using seven-point scales (valence: 1: extremely negative; 4: moderately positive; 7: extremely positive; and arousal: 1: extremely clam; 4: moderately arousing; 7: extremely arousing).

**Figure 1:**
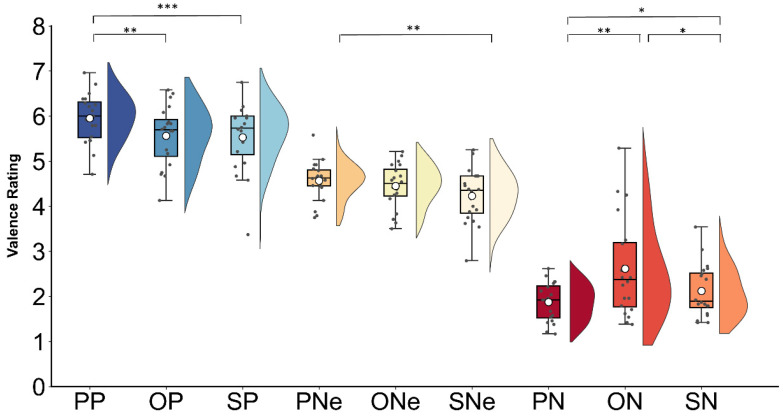
Mean valence ratings for each type of sentences (PP—partner positive; OP—opponent positive; SP—stranger positive; PNe—partner neutral; ONe—opponent neutral; SNe—stranger neutral; PN—partner negative; ON—opponent negative; and SN—stranger negative). Boxes represent the upper and lower quartiles, the solid lines indicate the median values, and the white points represent the mean values. The gray points on the graph correspond to the mean rating scores provided by each participant. The asterisks (*) denote a statistically significant effect (* *p* ≤ 0.05, ** *p* ≤ 0.01, *** *p* ≤ 0.001).

Results of two-way repeated measures ANOVA on valence and arousal ratings were presented. For the valence rating, significant main effects of interpersonal distance (*F*(1,19) = 7.65, *p* = 0.02, *η_p_*^2^ = 0.28) and contextual valence (*F*(1,19) = 221.99, *p* < 0.001, *η_p_*^2^ = 0.92) were observed. Follow-up tests showed that in comparison to the stranger-related sentences (M ± SE, 3.95 ± 0.09), the partner- (4.13 ± 0.07, *p* = 0.037) and opponent-related sentences (4.20 ± 0.09, *p* = 0.012) were rated as more pleasant, with no significant difference between the latter two conditions (*p* = 0.53). Moreover, the positive (5.68 ± 0.13) sentences were rated as more pleasant relative to the negative (2.20 ± 0.14, *p* < 0.001) and neutral sentences (4.41 ± 0.10, *p* < 0.001), and the neutral sentences were rated as more pleasant than the negative ones (*p* < 0.001). A significant interaction between interpersonal distance and contextual valence was found (*F*(1,19) = 9.93, *p* < 0.001, *η_p_^2^* = 0.34) (Figure 1). The post hoc tests showed that compared to the opponent-related (2.61 ± 0.24) and stranger-related (2.12 ± 0.13) sentences, the partner-related sentences were rated as more unpleasant (1.87 ± 0.96, *ps* < 0.033) in the negative context, while positive partner-related sentences (5.95 ± 0.12) were rated as more pleasant in the positive context compared to opponents (5.56 ± 0.15, *p* = 0.007) and strangers (5.52 ± 0.16, *p* < 0.001). Moreover, in the neutral context, the partner-related sentences (4.56 ± 0.09) were rated as more pleasant than those in the stranger-related condition (4.23 ± 0.13, *p* = 0.003).

**Figure 2:**
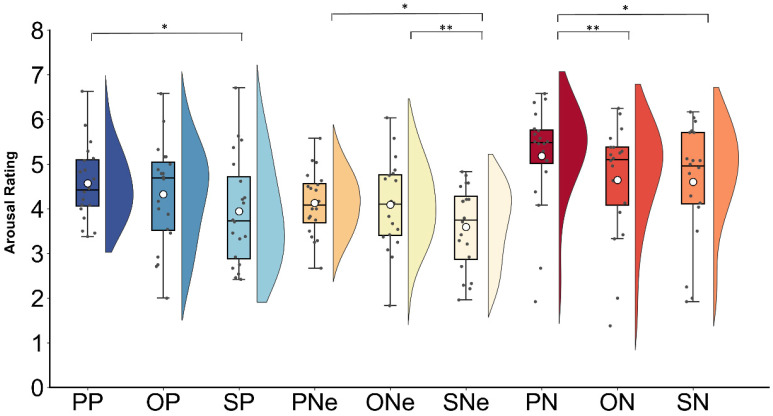
Mean arousal ratings for each type of sentence (PP—partner positive; OP—opponent positive; SP—stranger positive; PNe—partner neutral; ONe—opponent neutral; SNe—stranger neutral; PN—partner negative; ON—opponent negative; and SN—stranger negative). Boxes indicate the upper and lower quartiles, the solid lines indicate the median values, and the white points represent the mean values. The gray points on the graph correspond to the mean rating scores provided by each participant. The asterisks (*) denote a statistically significant effect (* *p* ≤ 0.05, ** *p* ≤ 0.01).

Regarding arousal, the main effects of interpersonal distance (*F*(1,19) = 9.58, *p* < 0.001, *η_p_*^2^ = 0.33) and contextual valence (*F*(1,19) = 10.39, *p* < 0.001, *η_p_*^2^ = 0.35) were both significant. Specifically, the partner-related sentences (4.62 ± 0.16) were rated as more arousing than the opponent-related (4.35 ± 0.23, *p* = 0.031) and stranger-related (4.04 ± 0.23, *p* = 0.004) sentences, but there was no significant difference between the latter two (*p* = 0.11). The positive (4.80 ± 0.26) and the negative (4.27 ± 0.22) sentences were rated as more arousing than the neutral ones (3.93 ± 0.18, *p*s ≤ 0.049), whereas the former two conditions showed no significant difference (*p* = 0.11). Furthermore, the interaction between interpersonal distance and contextual valence was also significant (*F*(1,19) = 4.09, *p* = 0.005, *η_p_*^2^ = 0.17), as shown in Figure 2. Post hoc tests showed that in the negative context, the partner-related sentences (5.17 ± 0.26) were rated as more arousing than the opponent-related (4.64 ± 0.28, *p* = 0.002) and stranger-related sentences (4.60 ± 0.29, *p* = 0.017). In the positive context, partner-related (4.57 ± 0.18) sentences exhibited greater arousal than the stranger-related ones (4.32 ± 0.26, *p* = 0.012). However, in the neutral context, both the partner-related (4.13 ± 0.16) and opponent-related sentences (4.09 ± 0.22) were rated as more arousing than the stranger-related ones (3.59 ± 0.20, *ps* < 0.015).

### 2.3. Procedure

The experiment was carried out in a chamber that was acoustically isolated and softly illuminated. Participants were seated approximately 70 cm away from a 19-inch monitor with the resolution of 1440 × 900 pixels (refresh rate, 60 Hz). The stimuli presentation and data collection were programmed in E-Prime software (Version 2.0, Psychology Software Tools, Inc., Pittsburgh, PA, USA).

**Figure 3:**
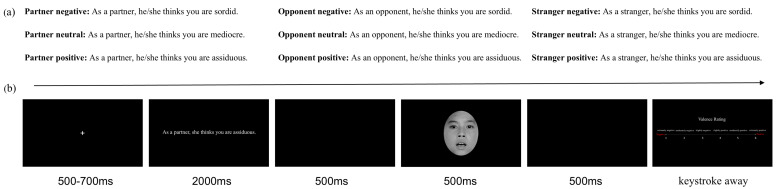
(**a**) Examples of contextual sentences. (**b**) Representative experimental trial.

Before the experiment, participants were instructed to envision themselves participating in a debate competition. Their partner served as their teammate, while the term ‘opponent’ referred to the debater representing the opposing team. The audience members who observed and evaluated the debate were referred to as ‘strangers’. The partner, opponent, and strangers in this context would all assess them, and the subsequent surprised face would belong to those individuals who conducted the evaluations. As shown in Figure 3b, each trial began with a white fixation cross for 500–700 ms, followed by a 2000 ms contextual sentence appearing at the center of the screen, during which participants viewed passively. A blank screen was then presented for 500 ms. Next, a 500 ms surprised face was displayed followed by a 500 ms blank screen. Finally, the rating scale screen was presented without time constraint, during which participants judged the valence of the surprised face on a 6-point scale via key pressing as quickly as possible (−3: extremely negative; −2: moderately negative; −1: slightly negative; 1: slightly positive; 2: moderately positive; 3: extremely positive). The response screen disappeared upon button pressing, and the next trial began after a 600 ms inter-trial blank screen.

To ensure a clear understanding of the task, participants will finish 18 trials for practice. In the main experiment, there are three blocks, each consisting of 144 trials. The block order was randomized, and in each block, three types of contextual valence sentences associated with a particular interpersonal distance were displayed in a counterbalanced order.

### 2.4. EEG Recording and Data Preprocessing

The EEG data were continuously recorded from 64 tin electrodes mounted on an elastic cap in accordance with the extended 10–20 system (Brain Products, Munich, Germany). The FCz electrode was used as the online reference. The recording sampling rate was set as 1000 Hz for each channel. Vertical electrooculograms (VEOG) were recorded from an electrode positioned 10 mm below the right eye. Electrode impedances of all electrodes were kept lower than 5 kΩ. Continuous EEG signals were filtered with a band-pass filter of 0.01–100 Hz.

The EEG data were analyzed offline using EEGlab (version 2023.0) [69] and ERPlab (version 8.01) [70] toolboxes implemented in MATLAB R2022b. The raw data were re-referenced to the average of all channels and filtered with a 0.01–30 Hz bandpass filter. Before average reference, channels with consistent noise were interpolated using the EEGlab’s multivariate local weighted regression tool. The mean number of interpolated electrodes per participant was 4.71 ± 1.27 (M ± SD). Independent component analysis (ICA; EEGlab “runica” function) was employed to correct various artifacts, such as eye blinks, eye movements, and muscle-related signals. Subsequently, the data were segmented into epochs, spanning from 200 ms before to 800 ms after the onset of surprised face. After baseline correction (−200 ms to 0 ms), epochs containing artifacts exceeding ±80 µV were automatically detected and excluded. On average, the partner-negative condition consisted of 46.09 ± 3.09 (M ± SD) trials, the partner-positive condition consisted of 46.14 ± 2.90 trials, the partner-neutral condition consisted of 46.09 ± 2.96 trials, the opponent-negative condition consisted of 46.51 ± 2.85 trials, the opponent-positive condition consisted of 46.63 ± 2.95 trials, the opponent-neutral condition consisted of 46.57 ± 2.67 trials, the stranger-negative condition consisted of 46.74 ± 2.36 trials, the stranger-positive condition consisted of 46.71 ± 2.46 trials, and the stranger-neutral condition consisted of 46.40 ± 2.18 trials for further signal averaging.

### 2.5. Data Analysis

***Behavioral analysis.*** Valence ratings were analyzed using SPSS Statistics 25. A two-way repeated measures analysis of variance (ANOVA) was used to investigate the effects of contextual valence and interpersonal distance.

***Factorial mass univariate analysis (ERP).*** Traditional ERP statistical analyses, focusing on peak or mean amplitudes at specific electrodes within predefined time windows, are susceptible to high type I or II error rates [70]. In contrast, the recently developed nonparametric mass univariate analysis performs separate statistical tests across all time windows and electrodes [71]. This approach mitigates the limitations of previous studies, leading to highly replicable and reliable experimental results. Therefore, we employed the mass univariate method to analyze the ERP data using the Factorial Mass Univariate Toolbox (FMUT) [71], which is an extension of the Mass Univariate Statistics toolbox. The FMUT calculates for the data of all timepoints and electrodes and conducts multiple comparisons through the permutation-based cluster mass technique [72,73]. The initial step involved conducting an exploratory ANOVA (α = 0.05) on all electrodes and time points ranging from 0 to 800 ms. Subsequently, the repeated-measures ANOVAs (α = 0.05) were performed to examine the priori time windows and regions of interest, including the occipito-temporal sites (P7, P8, PO7, PO8, O1, O2,) within the P1 (90–130 ms) and EPN (256–305 ms) time windows, occipito-temporal sites (P7, P8, PO7, PO8) within the N170 (140–190 ms) time window, and centro-parietal sites (C1, Cz, C2, CP1, CPz, CP2) within the LPP (400–600 ms) time windows. The time windows were selected slightly broader so that the targeted analysis would benefit from the data-driven approach without significantly compromising power [71]. Interpersonal distance and contextual valence were used as the within-subject factors. Then, follow-up ANOVAs were carried out to further explore the simple effects, with a Bonferroni-corrected α level of 0.016 (0.05/3 comparisons). Permutation-based cluster mass tests were employed for multiple comparisons correction in the ANOVAs [73]. Within the FMUT analysis, 100,000 permutations were performed for each data point [49].

## 3. Results

### 3.1. Behavioral Results

**Figure 4:**
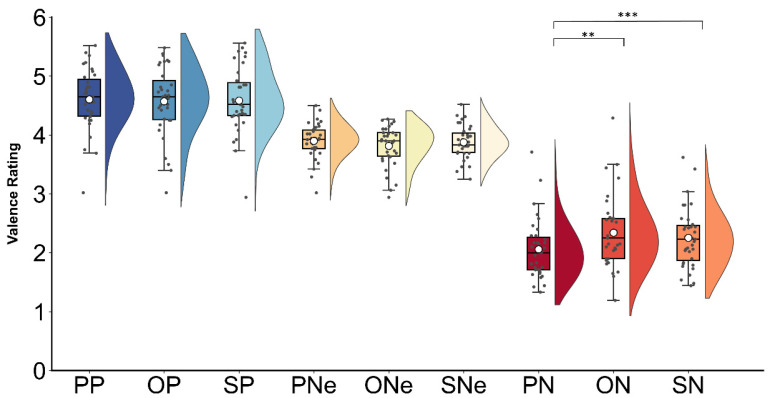
Mean valence ratings for surprised faces in different contexts (PP—partner positive; OP—opponent positive; SP—stranger positive; PNe—partner neutral; ONe—opponent neutral; SNe—stranger neutral; PN—partner negative; ON—opponent negative; and SN—stranger negative). Boxes indicate the upper and lower quartiles, the solid lines depict the median values, and the white points represent the mean values. The gray points on the graph correspond to the mean rating scores provided by each participant. The asterisks (*) denote a statistically significant effect (** *p* ≤ 0.01, *** *p* ≤ 0.001).

A significant main effect of contextual valence was found (*F*(2, 68) = 271.85, *p* < 0.001, *η_p_*^2^ = 0.86), with surprised faces in the positive contexts (M ± SE, 4.58 ± 0.08) rated as more pleasant compared to those faces in the neutral (3.86 ± 0.04, *p* < 0.001) and negative contexts (2.21 ± 0.08, *p* < 0.001), and surprised faces in the negative contexts rated as more unpleasant than those in the neutral contexts (*p* < 0.001). Furthermore, the interaction between interpersonal distance and contextual valence (*F*(4, 136) = 7.31, *p* < 0.001, *η_p_*^2^ = 0.17) also reached significance. As shown in Figure 4, surprised faces were rated as more unpleasant when presented in the negative partner contexts (2.05 ± 0.08) than in the negative opponent (2.34 ± 0.10, *p* = 0.003) and stranger (2.25 ± 0.08, *p* = 0.001) contexts, but the rating differences across other conditions were not significant (*ps* > 0.22). No significant main effect of interpersonal distance was found (*F*(2, 68) = 1.27, *p* = 0.28, *η_p_*^2^ = 0.03).

### 3.2. ERP Results

#### 3.2.1. Exploratory Analysis over All Electrodes (0–800 ms)

**Figure 5:**
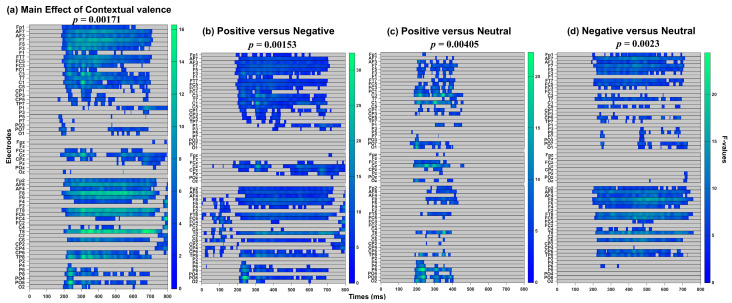
(**a**) The exploratory analysis was conducted within a time frame of 0–800 ms, encompassing all electrodes. The follow-up comparisons include (**b**) positive versus negative conditions, (**c**) positive versus neutral conditions, and (**d**) negative versus neutral conditions. The permutation-based cluster mass technique was employed to correct for multiple comparisons, with a significance level of *p* < 0.05 for the main effect and *p <* 0.016 for the post hoc paired comparisons.

The result showed a significant main effect of contextual valence from approximately 164–800 ms encompassing P1, EPN, and LPP components (Figure 5a; Table 1), with the maximal effect on FC4 at 792 ms (*F*(2, 68) = 14.89, *p* = 0.001). Follow-up ANOVAs (*p*-value threshold of 0.016) suggested that this was driven by differences between the surprised face in positive and negative contexts peaking at C1 around 308 ms (*F*(1, 34) = 23.97, *p* = 0.001; Figure 5b), in positive and neutral contexts peaking at T8 around 402 ms (*F*(1, 34) = 21.36, *p* = 0.004; Figure 5c), and in negative and neutral contexts peaking at PO7 around 120 ms (*F*(1, 34) = 19.15, *p* = 0.002; Figure 5d). However, there was no significant main effect of interpersonal distance (*p* = 0.27) or the interaction between interpersonal distance and contextual valence (*p* = 0.17).

#### 3.2.2. P1 Component over Occipito-Temporal Sites (90–130 ms)

**Figure 6:**
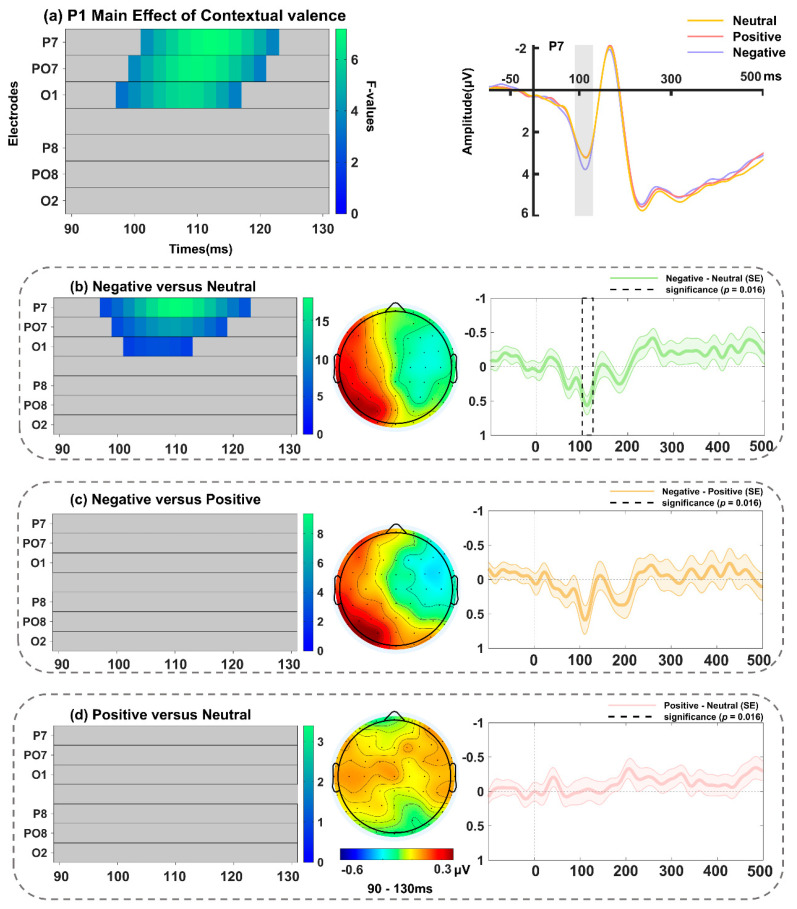
(**a**) Left: Main effect of contextual valence during the P1 time window (90–130 ms). Right: Grand-averaged ERP waveforms of P1 for surprised faces in positive (pink line), neutral (yellow line), and negative (purple line) contexts at electrode of P7. Follow-up comparisons between (**b**) negative versus neutral conditions, (**c**) negative versus positive conditions, and (**d**) positive versus neutral conditions in FMUT are displayed. The middle panel indicates the corresponding difference topographical maps during the P1 time range. The right panel illustrates the corresponding difference waveforms at P7, the dashed box denotes the significant time window observed in the analysis, and the shaded areas represent the standard error of the mean ERP amplitude across subjects.

**Figure 7:**
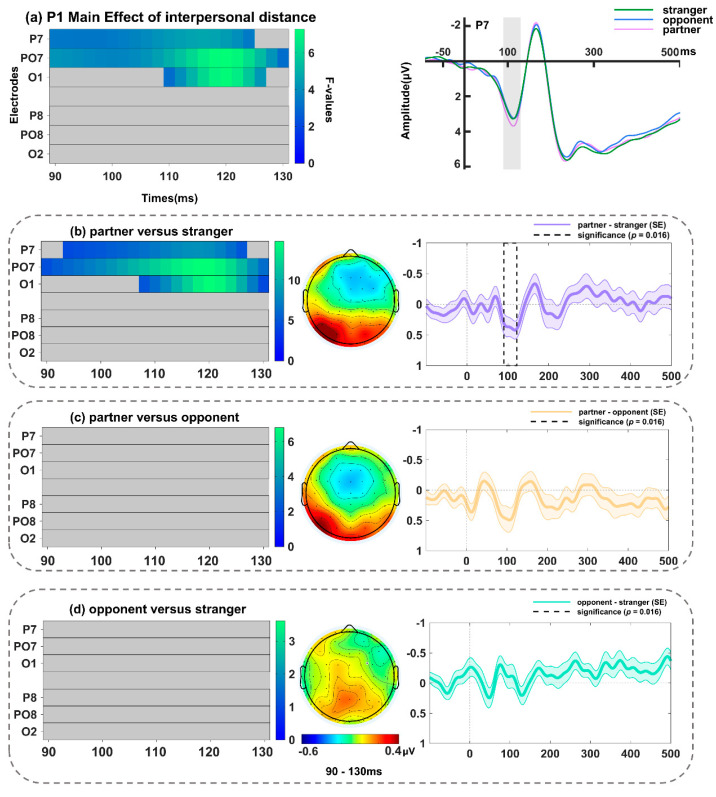
(**a**) Left: Main effect of interpersonal distance during the P1 time window (90–130 ms). Right: Grand-averaged ERP waveforms of P1 for surprised faces in partner-related (light purple line), opponent-related (dark blue line), and stranger-related (dark green line) contexts at P7. Follow-up comparisons between (**b**) partner versus stranger conditions, (**c**) partner versus opponent conditions, and (**d**) opponent versus stranger conditions in FMUT are displayed. The middle panel indicates the corresponding difference topographical maps during the P1 time range. The right panel illustrates the corresponding difference waveforms at P7, the dashed box denotes the significant time window observed in the analysis, and the shaded areas represent the standard error of the mean ERP amplitude across subjects.

There was a significant main effect of contextual valence in the electrodes of P7, PO7, O1 (Figure 6a and Table 2), peaking at P7 around 112 ms (*F*(2, 68) = 7.17, *p* = 0.015). The follow-up tests (spanning 90–130 ms; including electrodes P7, PO7, and O1, with a *p*-value threshold of 0.016) showed that the P1 amplitude elicited by surprised faces in the negative context was significantly larger than those in the neutral (greatest effect at P7, *F*(1, 34) = 17.92, *p* = 0.009; Figure 6b) contexts. In addition, a significant main effect of interpersonal distance in the electrodes of P7, PO7, and O1 was noted (Figure 7a and Table 2), peaking at PO7 around 120 ms (*F*(2, 68) = 7.27, *p* = 0.009). Further analyses (spanning 90–130 ms; including electrodes P7, PO7, and O1, with a *p*-value threshold of 0.016) revealed an enhanced P1 amplitude in response to surprised faces in the partner relative to stranger context (greatest effect at PO7, *F*(1, 34) = 14.70, *p* = 0.003; Figure 7b). The interactions between interpersonal distance and contextual valence were not significant (no clusters found).

#### 3.2.3. N170 Component over Occipito-Temporal Sites (140–190 ms)

Neither main effects of interpersonal distance (no clusters found) and contextual valence (*p* = 0.08) nor their interaction (no clusters found) were observed.

#### 3.2.4. EPN Component over Occipito-Temporal Sites (256–305 ms)

**Figure 8:**
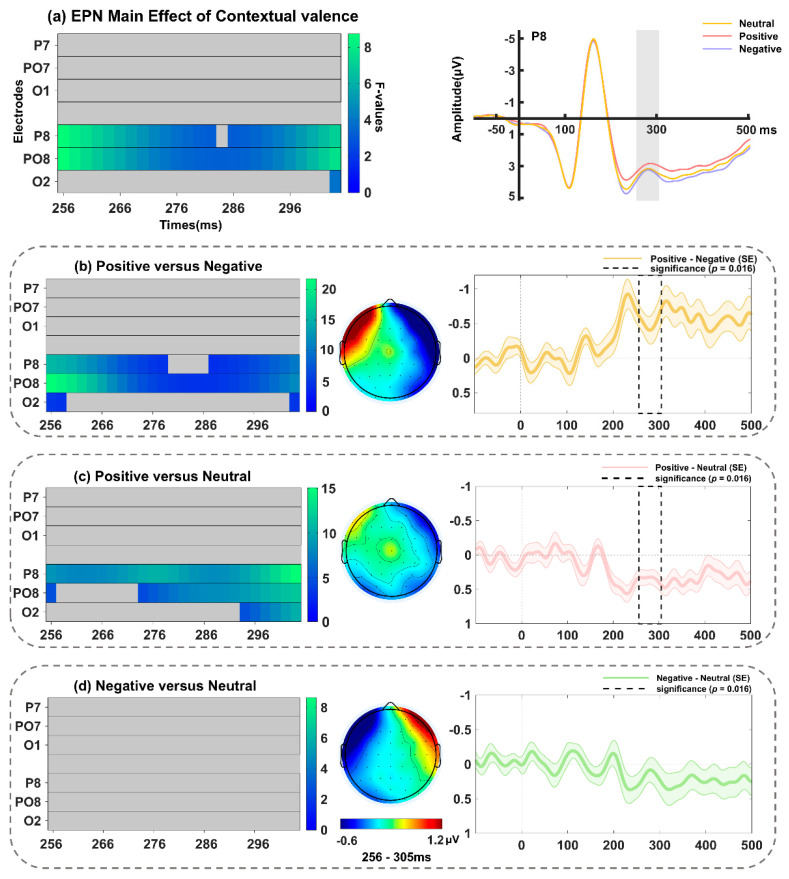
(**a**) Left: Main effect of contextual valence on the EPN (256–305 ms). Right: Grand-averaged ERP waveforms of EPN for surprised faces in positive (pink line), neutral (yellow line), and negative (purple line) contexts at the representative P8 electrode. Follow-up comparisons between (**b**) positive versus negative conditions, (**c**) positive versus neutral conditions, and (**d**) negative versus neutral conditions in FMUT are displayed. The middle panel indicates the corresponding difference topographical maps during the EPN time range. The right panel illustrates the corresponding difference waveforms at P8, the dashed box denotes the significant time window observed in the analysis, and the shaded areas represent the standard error of the mean ERP amplitude across subjects.

**Figure 9:**
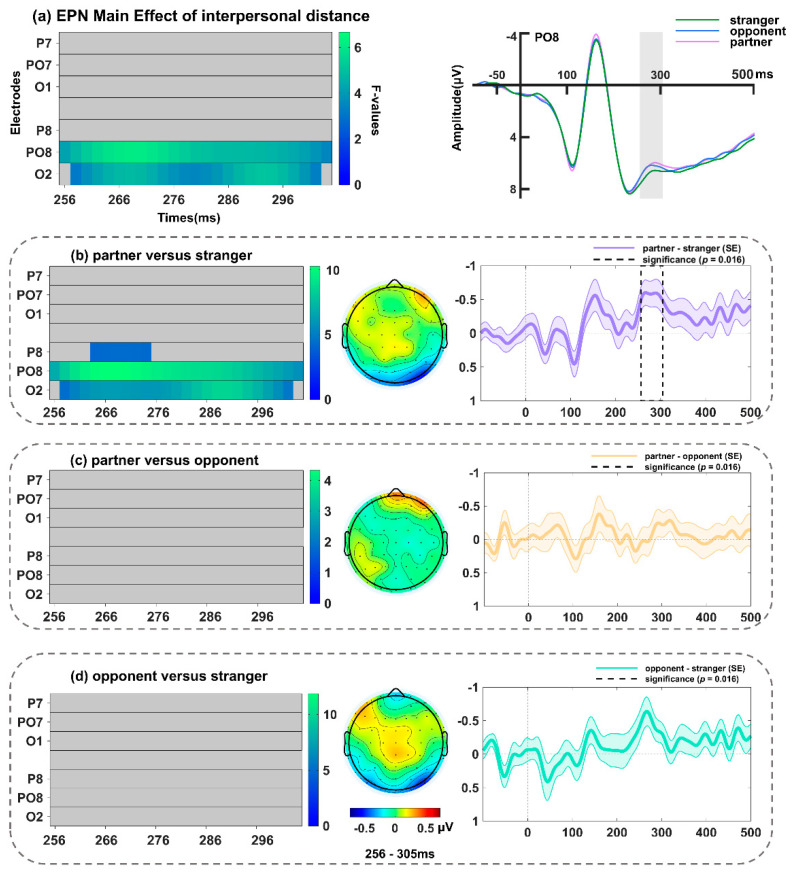
(**a**) Left: Main effect of interpersonal distance on the EPN (256–305 ms). Right: Grand-averaged ERP waveforms of EPN for surprised faces in partner-related (light purple line), opponent-related (dark blue line), and stranger-related (dark green line) contexts at PO8. Follow-up comparisons between (**b**) partner versus stranger conditions, (**c**) partner versus opponent conditions, and (**d**) opponent versus stranger conditions in FMUT are presented. The middle panel indicates the corresponding difference topographical maps during the EPN time range. The right panel illustrates the corresponding difference waveforms at PO8, the dashed box denotes the significant time window observed in the analysis, and the shaded areas represent the standard error of the mean ERP amplitude across subjects.

The analysis revealed a main effect of contextual valence that encompassed P8, PO8, and O2 (Figure 8a and Table 2), and peaked at PO8 around 256 ms (*F*(2, 68) = 8.72, *p* = 0.013). Further analysis (spanning 230–310 ms; including electrodes P8, PO8, and O2, with a *p*-value threshold of 0.016) revealed an increased amplitude of the EPN in response to surprised faces in the positive relative to negative (peaking at PO8, *F*(1, 34) = 21.73, *p* = 0.013; Figure 8b) and neutral (peaking at P8, *F*(1, 34) = 15.10, *p* = 0.007; Figure 8c) contexts, while there was no significant difference between the latter two conditions (*p* = 0.17). Furthermore, a significant main effect of interpersonal distance was identified, as one cluster encompassing P8, PO8, and O2 (Figure 9a and Table 2), and the maxima was found at 268 ms on PO8 (*F*(2, 68) = 6.08, *p* = 0.019). Follow-up tests (spanning 256–305 ms; including electrodes P8, PO8, and O2, with a *p*-value threshold of 0.016) revealed that the EPN amplitudes elicited by surprised faces in the partner context were larger than those in the stranger context (peaking at PO8, *F*(1, 34) = 10.24, *p* = 0.015; Figure 9b). Nevertheless, the interaction between interpersonal distance and contextual valence did not reach statistical significance (*p*s > 0.08).

#### 3.2.5. LPP Component over Centro-Parietal Sites (400–600 ms)

**Figure 10:**
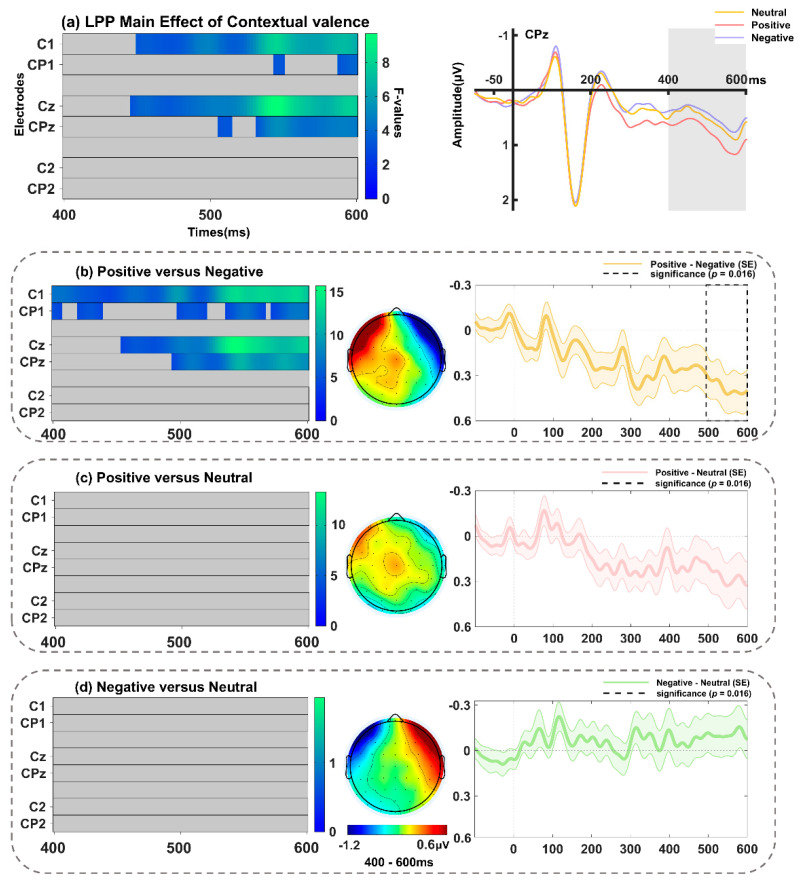
(**a**) Left: Main effect of contextual valence on the LPP (400–600 ms). Right: Grand-averaged ERP waveforms of LPP for surprised faces in positive (pink line), neutral (yellow line), and negative (purple line) contexts at the representative electrode of CPz. Follow-up comparisons between (**b**) positive versus negative conditions, (**c**) positive versus neutral conditions, and (**d**) negative versus neutral conditions in FMUT are displayed. The middle panel indicates the corresponding difference topographical maps during the LPP time range. The right panel illustrates the corresponding difference waveforms at CPz, the dashed box denotes the significant time window observed in the analysis, and the shaded areas represent the standard error of the mean ERP amplitude across subjects.

**Figure 11:**
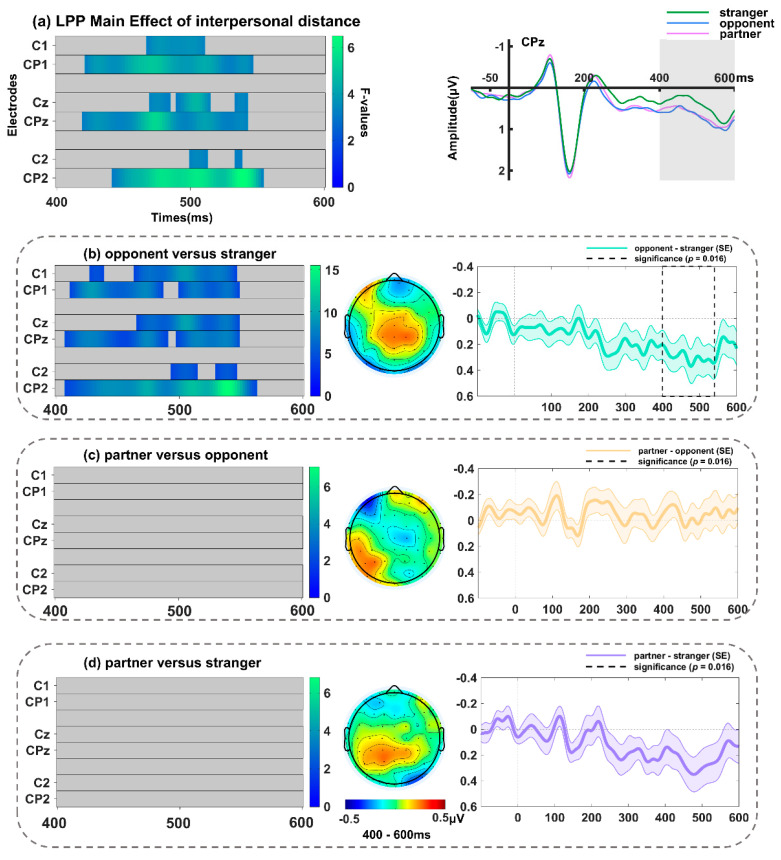
(**a**) Left: Main effect of interpersonal distance on the LPP (400–600 ms). Right: Grand-averaged ERP waveforms of LPP for surprised faces in partner-related (light purple line), opponent-related (dark blue line), and stranger-related (dark green line) contexts at the representative electrode of CPz. Follow-up comparisons between (**b**) opponent versus stranger conditions, (**c**) partner versus opponent conditions, and (**d**) partner versus stranger conditions in FMUT are displayed. The middle panel indicates the corresponding difference topographical maps during the LPP time range. The right panel illustrates the corresponding difference waveforms at the CPz, the dashed box denotes the significant time window observed in the analysis, and the shaded areas represent the standard error of the mean ERP amplitude across subjects.

The significant main effect of contextual valence was observed on C1, Cz, C2, CP1, CPz, and CP2, with the maximum value at Cz around 544 ms (Figure 10a and Table 2) (*F*(2, 68) = 9.68, *p* = 0.011). Subsequent examinations (spanning 400–600 ms; including electrodes C1, Cz, C2, CP1, CPz, and CP2, with a *p*-value threshold of 0.016) showed that the LPP amplitudes were enlarged for surprised faces in the positive relative to negative context, with the most significant effect at Cz (Figure 10b) (*F*(1, 34) = 15.51, *p* = 0.005), but no differences were found between the other conditions (*ps* > 0.032). Furthermore, the cluster encompassing C1, Cz, C2, CP1, CPz, and CP2 exhibited a significant main effect of interpersonal distance, which reached its peak at CP2 around 540 ms (Figure 11a and Table 2) (*F*(2, 68) = 6.77, *p* = 0.011). Further analyses (spanning 400–600 ms; including electrodes C1, Cz, C2, CP1, CPz, and CP2, with a *p*-value threshold of 0.016) indicated that the LPP amplitude elicited by surprised faces in the opponent-related context was enhanced as compared to those in the stranger-related context (most significant effect at CP2, *F*(1, 34) = 15.41, *p* = 0.007; Figure 11b), but the differences between other conditions were not significant (*ps* > 0.052). No significant interaction between interpersonal distance and contextual valence was found (*p* = 0.39).

## 4. Discussion

Considerable studies have demonstrated that the interpretation of facial expressions can be heavily biased by perceived contextual information [8,23,40,74]. Building upon these findings, the present study explored how and when contextual valence and interpersonal distance influence the perception of surprised faces over time. Behavioral results revealed a significant interaction between contextual valence and interpersonal distance, with surprised faces in partner-related negative contexts receiving more unpleasant ratings relative to those in opponent- and stranger-related negative contexts. This finding expands the understanding of contextual effects on face perception to the realms of cooperative and competitive relationships, suggesting a preference for processing negative stimuli. Previous research conducted in both human and animal subjects has consistently indicated that the utilization of negative reinforcement promoted faster learning speed in comparison to positive reinforcement, reflecting a robust negativity bias [75,76]. For higher-order cognitive processes, negative aspects of events or stimuli are considered to possess greater informational value compared to positive aspects, necessitating heightened attention allocation and elaborated processing [77,78]. Additionally, self-reference has been found to shape the perception of facial expressions [23]. People are better at recognizing facial expressions from their own group compared to other groups due to the ubiquitous own-group bias [79]. Partners, being considered part of our own group and holding a higher level of self-relevance than opponents and strangers, may therefore elicit stronger reactions. This is consistent with the findings of Rajchert et al. [66], which reported stronger negative affect and hurt feelings in response to rejection by friends compared to acquaintances and strangers. Moreover, negative judgements from partners may violate the expectation of acceptance in close relationships, triggering a heightened sense of threat to belonging and more intense hurt feelings [80,81]. This could explain the lower ratings of surprised faces in negative partner-related contexts, suggesting a potential self-protection mechanism at play.

### 4.1. Effect of Contextual Valence

At the electrophysiological level, pronounced contextual valence effects were observed in both the early and late stages of surprised face processing. Specifically, surprised faces presented in negative contexts elicited larger P1 amplitudes than those in neutral contexts. Numerous neuroimaging studies have demonstrated that the P1 component is involved in processing sensory features of exogenous stimuli in the extrastriate cortex [82,83], and the encoding of visual properties associated with affective saliency triggers a top-down neural signal in the frontal cortex and then boosts activations in the occipitotemporal region [84]. ERP studies on face perception also uncovered that the P1 is linked to the early detection of emotional facial expressions, exhibiting a processing advantage for threatening expressions [38,85,86]. Additionally, an individual’s prior experiences and environment could influence the P1 response to faces [87,88]. Typically, individuals initially interpret the valence of surprised faces negatively, indicative of an inherent negativity bias [89,90]. Li et al. [40] also observed enhanced P1 amplitudes for surprised faces following subliminal fear-inducing stimuli. Our results align with these findings, showing increased P1 amplitudes for surprised faces in negative sentence contexts, presumably reflecting enhanced attentional capture by threat-related stimuli. On the other hand, Hu and Liu [91] argued that emotional contexts not only trigger corresponding affective states but also generate appropriate anticipation about upcoming visual input. Aligning with this notion, studies have shown that anticipation can influence the even earlier C1 component, intensifying responses to surprised faces in both fearful and happy contexts [53]. This suggests that top-down anticipation triggered by preceding context may influence facial perception at an early sensory stage. Our findings seem to partially support this view, as a significant emotional context effect was only observed in the left hemisphere, indicating the hemispheric superiority in the perception of verbal stimuli [92]. Therefore, caution is required when interpreting the P1 emotion effect, as it may be associated with both bottom-up contextual valence and high-order expectation.

For the EPN, we observed a significant main effect of contextual valence, with increased amplitudes for surprised faces in positive compared to neutral and negative contexts, which supports our hypothesis. EPN has been shown to be sensitive to the arousal level of stimuli [55,93,94,95] and modulated by contextual information [6,8,23]. For instance, neutral or surprised faces in self-related positive contexts elicit larger EPN amplitudes than those in sender-related positive contexts [23,32], reflecting the contribution of self-positivity bias [26,96]. Self-positivity bias refers to the tendency of individuals to associate positive characteristics with themselves and negative characteristics with others [97], leading to amplified self-enhancement motivation. There is direct evidence that the influence of self-reference on visual stimuli processing starts at the EPN, with enlarged responses for the self-related condition [8,23,98,99]. The integration of self-related and emotional information in processing facial expressions is linked to numerous brain regions associated with the EPN, such as the temporal lobe, occipital-temporal cortex, amygdala, and fusiform gyrus [56,100,101]. Specifically, the visual cortex of the occipital lobe is implicated in selective attention and motivational processing of emotional information [101,102], while the fusiform face area (FFA) specifically contributes to self-face recognition [103]. This may partially explain why the EPN response is associated with both emotional and self-related information. In our study, all contextual sentences followed the pattern “He/She thinks you are optimistic”, which rendered the target faces highly self-relevant to the participants. Therefore, it is likely that surprised faces in a self-related positive context may carry higher emotional significance due to the role of self-positivity bias, resulting in more elaborative processing and increased brain activity.

The LPP component is known to be enhanced by emotionally valenced faces or even isolated eye regions [42,52,55,104]. Moreover, substantial evidence suggested that ambiguously valenced expressions presented in emotional contexts, especially threatening ones, can evoke larger amplitudes. This indicated that emotional contexts could increase the motivational significance of target faces in a top-down fashion [22,23,31,105]. Furthermore, a study on healthy adults combined ERP and fMRI methods, revealing that the presentation of emotional stimuli resulted in both an augmentation of the LPP and enhanced activations within the occipital, parietal, inferior temporal regions, as well as the dorsolateral prefrontal cortex [106]. Importantly, the dorsolateral prefrontal cortex may modulate the attention network in the parietal lobe, which is involved in processing motivationally salient stimuli [107]. Therefore, the increased LPP amplitude seems to reflect a heightened attentional allocation towards stimuli with motivational significance [108,109,110]. Here, we observed a stable effect of contextual valence on the LPP, with increased amplitudes for surprised faces in positive relative to negative contexts. This finding reaffirms the role of motivated attention [87,111,112]. Notably, Herbert et al. [112] found that negatively valenced stimuli quickly capture attention during the early perceptual stages, while positively valenced stimuli attract more selective attention for detailed representations during the mid-latency and late processing stages, which is termed the subsequent positivity offset effect [111,113]. Our results appear to support this view, indicating initial attentional engagement with negative contexts in the early P1 stage, followed by a progressive enhancement of attention toward surprised faces within positive contexts during the EPN and LPP stages.

### 4.2. Effect of Interpersonal Distance

Interestingly, an evident interpersonal distance effect has been noted throughout the early to late stages of processing surprised faces. Both the P1 and EPN exhibited enhanced amplitudes for surprised faces in partner- compared to stranger-related contexts, irrespective of emotional valence. For the P1, this finding aligns with the view that self-related stimuli and tasks can amplify the modulation of higher-order cognitive processes at the early sensory stages due to their inherent motivational significance [114,115]. Previous language research has found that, in some cases, preceding contextual cues could significantly amplify the top-down attentional effect within the P1 and N1 time ranges [116]. Rubinsten et al. [117] also proposed that close friends, rather than strangers, can rapidly capture attention during the N1 stage in healthy individuals. Likewise, subjects were presented with contextual sentences representing various interpersonal distances prior to the presentation of surprise faces in our experiment. They were instructed to imagine their partners as teammates who participated in the competition alongside them, while strangers were described as individuals unrelated to the competition, such as spectators. In this case, the amplified P1 responses to surprised faces in partner-related contexts might be attributed to the heightened self-relevance driven by top-down attention.

In terms of the EPN, several studies have revealed that cooperative interactions, as opposed to affective ones, in real-life settings can elevate the N2 amplitude [118], suggesting that cooperation enhances the motivational significance of stimuli. Furthermore, the EPN has been shown to vary as a function of self-relevance, exhibiting larger amplitudes for faces in contexts related to the self [23,31,33]. Echoing these discoveries, the partner contexts in our study also signal cooperative intentions and a higher degree of self-relevance. This could amplify the emotional or motivational significance of subsequent surprised faces to some extent, fostering increased selective attention and strategic encoding.

Unexpectedly but interestingly, we observed an enlarged response for surprised faces in opponent- relative to stranger-related contexts in the LPP time window. Neutral faces in negative rather than neutral contexts were associated with enhanced LPP responses [31], highlighting the impact of negative context on detailed face processing. Meanwhile, the relevance of self-interest may intensify the contrast in motivational values between positive and negative outcomes within the context of social comparison [119]. In our study, ‘opponents’ were depicted as fellow competitors, while ‘strangers’ were portrayed as mere spectators. Compared to strangers, the heightened relevance of shared interests with opponents suggests an implicit hostility. Consequently, opponent-related cues could recruit more attentional resources, thereby amplifying LPP responses. Additionally, numerous studies have consistently demonstrated the substantial impact of self-relevance on LPP, with increased responses for self-related stimuli [8,33]. As such, the heightened self-relevance associated with opponent contexts could account for the differential LPP responses observed here.

Overall, our use of the innovative FMUT method enhances the robustness and reproducibility of our findings by minimizing type I and type II errors and capturing subtle differences between conditions. Furthermore, our results reveal a nuanced shift in attentional focus from negative (P1) to positive (EPN, LPP) contextual cues during the processing of surprised facial expressions. Independent of contextual valence, partner-related contexts initially heighten early selective attention to target faces (P1, EPN), whereas opponent-related contexts elicit sustained attention and more elaborate face processing (LPP). These findings significantly advance our understanding about the temporal dynamics of processing ambiguous facial expressions within competitive/cooperative interpersonal and emotional contexts. By delving into this uncharted territory, our study not only expands the literature on the neural mechanisms underlying the processing of intricate social information but also sheds novel insights into the higher-order cognitive processes operating within competitive/cooperative contexts.

Some limitations of this study should be acknowledged. Prior research utilizing event-related designs to investigate the contextual effects of facial expression perception has identified significant interactions in mid-late components [6,23,31]. However, due to the complexity of the situational materials employed in this study, we presented interpersonal distance information in separate blocks. This approach may have facilitated participants’ acclimation to the interpersonal distance information, potentially leading to its disregard. Consequently, not only did this weaken its impact on the ERP results, but it could also account for the absence of a significant main effect of interpersonal distance on the behavioral ratings. Furthermore, traditional assessments of contextual material have predominantly focused on two dimensions: valence and arousal. Drawing from the existing literature [3,8,22,23], our study utilized valence and arousal as criteria for evaluating context, without directly assessing interpersonal distance. This methodological choice may have limited our ability to fully interpret the influence of interpersonal distance on ERPs. Future research should consider both experimental design and material assessment to thoroughly examine the stability and generalizability of the effects observed here.

In summary, this study offers valuable insights into the impact of interpersonal distance, as a form of self-related information, on the perception of ambiguous surprised faces within varying emotional contexts. The ERP findings suggest that these two types of social contextual information exert independent modulations on face processing in a top-down manner. Specifically, the effect of emotional valence is characterized by a transition from negative (P1) to positive attentional biases (EPN, LPP), while the influence of interpersonal distance also demonstrates a tendency to shift from positive (P1, EPN) to negative (LPP) responses, transitioning from early friendly messages to subsequent threatening messages.

## Figures and Tables

**Table 1:** Summary table of the main effect (*p* < 0.05) of exploratory analysis. This table includes the time windows of significance with their associated electrodes. The overall peak *p*- and *F*-value of the analyses are also provided.

Test	Timing	Electrodes	Peak
Contextual valence	164–768 ms	Fp2, F4, C4, P4, O1, O2, F8, T8, P7, P8, Oz, FC6, CP5, CP6, AF4, FC4, PO3, PO4, F6, C6, P5, P6, AF8, FT8, TP8, PO7, PO8, Fpz	PO8 at 238 ms [*F*(2, 68) = 16.24, *p* = 0.00188]
	178–800 ms	Fp1, F3, F4, C3, C4, P3, O1, F7, T7, P7, Cz, Pz, Oz, FC1, FC2, CP1, CP2, FC5, FC6, CP5, CP6, FCz, F1, F2, C1, C2, P1, AF3, AF4, FC3, FC4, CP3, CP4, PO3, F5, F6, C5, C6, P5, AF7, AF8, FT7, TP7, PO7, Fpz, CPz, POz	FC4 at 792 ms [*F*(2, 68) = 14.89, *p* = 0.00171]
Interpersonal distance	N/A	N/A	N/A
Contextual valence × Interpersonal distance	N/A	N/A	N/A

**Table 2:** Summary table of the main effect (*p* < 0.05) of prior analysis. This table includes the time windows of significance with their associated electrodes. The overall peak *p*- and *F*-value of the analyses are also provided.

ERPs	Test	Timing	Electrodes	Peak
P1 (90–130 ms)	Contextual valence	98–122 ms	O1, P7, PO7	P7 at 112 ms (*F*(2, 68) = 7.17, *p* = 0.0155)
	Interpersonal distance	90–130 ms	O1, P7, PO7	PO7 at 120 ms (*F*(2, 68) = 7.27, *p* = 0.00928)
N170 (140–190 ms)	Contextual valence	N/A	N/A	N/A
	Interpersonal distance	N/A	N/A	N/A
EPN (256–305 ms)	Contextual valence	256–304 ms	P8, PO8, O2	PO8 at 256 ms (*F*(2, 68) = 8.72, *p* = 0.01391)
	Interpersonal distance	256–304 ms	PO8, O2	PO8 at 268 ms (*F*(2, 68) = 6.08, *p* = 0.01997)
LPP (400–600 ms)	Contextual valence	446–600 ms	CP1, CPz, C1, Cz	Cz at 544 ms (*F*(2, 68) = 9.68, *p* = 0.0118)
	Interpersonal distance	420–554 ms	CP1, CP2, CPz, C1, C2, Cz	CP2 at 540 ms (*F*(2, 68) = 6.77, *p* = 0.01128)

## Data Availability

The data are available from the corresponding author upon reasonable request.
